# The Effect of Menstrual Cycle Phases on Approach–Avoidance Behaviors in Women: Evidence from Conscious and Unconscious Processes

**DOI:** 10.3390/brainsci12101417

**Published:** 2022-10-21

**Authors:** Danyang Li, Lepu Zhang, Xiaochun Wang

**Affiliations:** School of Psychology, Shanghai University of Sport, 650 Qing Yuan Huan Road, Yangpu District, Shanghai 200438, China

**Keywords:** menstrual cycle, approach–avoidance behaviors, estrogen, progesterone, conscious processing, unconscious processing, emotional stimuli

## Abstract

The menstrual cycle affects women’s emotional states, with estrogen and progesterone having predominant roles. However, it remains unclear whether the phases of the menstrual cycle also affect women’s motivational behaviors. In this study, the main aim was to investigate how the menstrual cycle influences approach–avoidance behavior under conditions of conscious versus unconscious processing of emotions. Briefly, after recruitment by advertisement and screening with a menstrual cycle survey questionnaire, 27 naturally cycling, healthy women participated in an improved “manikin task” and were presented both positive and negative emotional stimuli during early follicular, late follicular, and mid-luteal phases. Estrogen and progesterone levels were measured. Women in the late follicular phase exhibited the shortest response times for approaching positive stimuli, while women in the mid-luteal phase exhibited the shortest response times for avoiding negative stimuli. Estrogen and progesterone levels significantly correlated with the speed of the approach–avoidance responses observed for the women, indicating the important role that sex hormones have in mediating emotionally motivated behavior. Overall, these findings suggest that the menstrual cycle has strong and specific influences on women’s approach–avoidance behaviors that are in part mediated by estrogen and progesterone. By identifying characteristics of these behaviors in the late follicular and mid-luteal phases, greater insight can be provided to women regarding the physiological influences of the menstrual cycle on their personal growth and security.

## 1. Introduction

It is the instinct of creatures, and human nature, to approach advantages and avoid disadvantages. Profit seeking enables organisms to acquire stronger survival abilities, while avoidance of harm enables individuals to continue their lives. Each day, we make decisions regarding approach or avoidance behaviors and act accordingly. For example, we will smell a flower to enjoy its fragrance, yet will avoid a spider making a web. Both animals and humans naturally exhibit behavioral motivations toward obtaining rewards and avoiding threats, and these actions are related to behavioral approach and behavioral inhibition systems, respectively [1]. Many studies have investigated the relationship between emotional valence dimensions and behaviors [2,3,4]. To date, the results of these studies have consistently supported that a “compatible effect of approach–avoidance” (e.g., the “Affective Simon effect”) exists with conscious processing of emotional valence as a stimulus. This effect is also known as, “stimulus–response compatibility” (SRC), which refers to positive emotions facilitating approach behaviors and negative emotions contributing to avoidance behaviors. However, when individuals unconsciously process the emotional valence of stimuli, this effect has not been observed [4,5].

The Motivational Orientation Theory and the Theory of Event Coding (TEC) both try to explain the phenomenon of “compatible effect of approach–avoidance”, yet they encompass different opinions. The former [6,7] suggests that emotional stimuli can activate a motivation consistent with stimulus valence without explicit processing of the stimulus valence. In contrast, the TEC [6] proposes that both approach behavior and positive stimuli have positive valence, while avoidance behavior and negative stimuli have negative valence. A compatibility effect is caused by a consistent valence of positive and negative stimuli and approach–avoidance responses. It has been observed that a compatibility effect disappears when a task involves implicit emotion processing, that is, when explicit valence judgments are not involved. Zhang et al. (2012) analyzed differences between these two theories by mainly focusing on whether compatible approach–avoidance behaviors can be automatically (unconsciously) activated without relying on explicit emotional evaluation [8].

There have been two studies that support the Motivational Orientation Theory. Alexopoulos and Ric (2007) created implicit emotional processing conditions by presenting emotional stimuli subliminally [9]. Subsequently, Krieglmeyer et al. (2010) conducted an experimental study that asked subjects to judge the grammatical category of emotional words [3]. This effect was found in both unconscious processing processes. Regarding the TEC, Rotteveel and Phaf (2004) asked subjects to make approach (arm bending) and avoidance (arm extension) responses to a valence of facial expressions or gender information [4]. When the subjects were asked to judge the valence of faces, a “compatible effect of approach–avoidance” was observed. However, when the task was to judge the gender of faces, no effect was observed. Currently, there remains a great debate between these two theories. Researchers mostly believe that the “push-pull rod task” conducted by Rotteveel and Phaf (2004), which involves a deliberately manipulated arm movement, does not clearly represent the approach–avoidance tendency [4]. Furthermore, this task is not sensitive enough to represent emotional information [10]. Consequently, the “Manikin Task” paradigm is commonly used to investigate the SRC effect. In this paradigm, the emotional valence (positive and negative) of a stimulus and the label valence (upward and downward) of the approach–avoidance response can be effectively separated. This aspect has advantages over other related paradigms in terms of sensitivity and criterion validity [3,11]. In addition, more recent experiments have used emotional pictures to induce the corresponding emotions of the subjects, and the self-face of each participant is used as a unique self-reference stimulus instead of a manikin. The latter approach is referred to as an improved “manikin task” [12]. Despite its use, however, the improved “Manikin Task” paradigm has not been used to explore whether compatible, approach–avoidance behaviors require the intervention of consciousness.

An active area of research in the field of mood studies is the role of sex hormones. One possible explanation for the differences in emotional processing observed in males versus females often involves the changes in ovarian hormone levels that characterize a woman’s menstrual cycle. Previous studies have shown that changes in hormonal levels during the female menstrual cycle can affect women’s emotions, cognition, and social behaviors [13]. In particular, roles for estradiol and progesterone in emotional behavior have been demonstrated [14,15]. The menstrual cycle refers to the periodic shedding and bleeding of a woman’s endometrium. The physiological hormones in a woman’s body differ during the various phases of the menstrual cycle. For example, both progesterone and estradiol are present at their lowest levels during menstruation, while estradiol levels rise during ovulation. The latter peaks in the late follicular phase, while progesterone levels remain low. In the early and middle luteal phase, progesterone is at its highest level, while estradiol is at an intermediate level. In the late luteal phase, progesterone and estradiol are both at low levels. It has been confirmed that estradiol and progesterone play important roles in the psychological state experienced during the various phases of menstrual cycles. Patients were consistently more restless, irritable, fatigued, fearful, and depressed during the premenstrual period than other phases of the menstrual cycle, as well as being hypersensitive to various stimuli [16]. Dreher (2007) et al. found that both expected and actual monetary rewards caused higher activation of the reward system in mid-follicular, and this response was related to estrogen levels [17]. In contrast, progesterone in the luteal stage will enhance women’s response to negative emotions [18,19]. Other researchers have specifically explored how the prioritization of visual emotional stimuli is affected by menstrual cycle stage. The results obtained suggest that sex hormones influence the processing of visual threat cues in a social context. Specifically, women in the luteal phase are more sensitive to infectious and threatening faces containing negative information and have an increased heart rate during observation than women in the late follicular phase [20]. Processing of non-social threatening stimuli also differs in different phases of the menstrual cycle [21]. For example, in the luteal phase, women have shorter responses to threatening stimuli (e.g., snakes, tigers, and other predators) than in the early and late follicular phases. However, when presented with neutral stimuli (e.g., plants, etc.), no difference in response time between cycles is observed, suggesting that increased alertness during the luteal phase is selective and is only related to emotional context. In addition, during the luteal phase, women are more likely to exhibit food aversions, especially if a given food may be the source of pathogens (e.g., meat; reviewed in [22]).

### 1.1. Aims

Accumulating evidence has demonstrated that during the follicular and luteal phases, women exhibit different physical and psychological manifestations in response to positive and negative emotional stimuli, and these are closely related to secretion of the hormones estradiol and progesterone. While many studies have explored emotional valence during the female menstrual cycle, only a small number of studies have examined emotional motivation during the female menstrual cycle. According to changes in the external environment or events, people adjust the activation level of their motivation system and implement approach or avoidance behaviors. These responses are of great significance for individual survival and development. Moreover, especially for women who are increasingly in need of promotion and are more vulnerable to security threats, their motivational behaviors in response to positive or negative external information are very important for them. Therefore, it is particularly important to understand whether women’s motivation to seek advantages and avoid disadvantages is affected by the menstrual cycle and its pattern of changes. In addition, the dispute between motivational orientation and event-coding theories needs to address considerations of more potent paradigms. We conducted the present study to help women better understand their bodies and minds and to contribute to and provide support for women’s safety and emotional needs.

### 1.2. Plan and Hypothesis

Briefly, we conducted two experiments. The purpose of Experiment 1 was to determine the specific performance of the “compatible effect of approach–avoidance” in women’s conscious processing at different phases of their menstrual cycle. Experiment 2 was designed to examine whether women also exhibit the “compatible effect of approach–avoidance” during unconscious processing, and how this effect is affected by the menstrual cycle. To evaluate the influence of conscious processing, approach–avoidance responses to emotional valence were observed. To evaluate the influence of unconscious processing, approach–avoidance responses to the presence or absence of a person in an emotional picture were observed. We made assumptions that women in the late follicular phase exhibited a shorter response time to positive stimuli than women in the early follicular phase. Conversely, women in the mid-luteal phase exhibited a shorter response time in avoiding negative stimuli than women in the late follicular phase. Estradiol and progesterone played an important role in the specific approach–avoidance behavior observed for the women.

## 2. Methods

### 2.1. Participants

A total of 27 female college students were included in this study. They were all right-handed and had an average age of 22.78 (±2.35; range, 19–28). The menstrual cycles of all the potential participants were monitored using the Prospective Record of the Impact and Severity of Menstrual Symptoms (PRISM) [23] before they were enrolled in this study. The PRISM calendar presents emotional (e.g., depression) and behavioral (e.g., insomnia), as well as daily physical symptoms (e.g., breast tenderness), which are graded by severity (asymptomatic, mild, moderate, and severe). Potential participants were asked to complete daily entries in a calendar spanning three consecutive menstrual cycles. Potential participants also completed a menstrual cycle survey questionnaire. Inclusion criteria for participants in this study were: a stable menstrual cycle length that ranged between 26 and 35 days over the preceding three months, no intake of any hormone drugs (including short-term and long-term contraceptives), no pregnancy plans within the subsequent six months, and no pregnancy within the previous six months. After completing all of the trials, the participants’ menstrual cycles were followed for one additional month to determine the stability of their menstrual cycles.

The reverse calculation method was employed to predict the experimental window of the late follicular and mid-luteal phases for each subject. The reliability of this method has previously been reported [24,25,26,27,28]. According to this method, taking a 28-day menstrual cycle as the standard, the day that menses begins is considered as the first day of the menstrual cycle, −15 to −17 days are the late follicular period, −7 to −9 are the mid-luteal period, and days 2–5 after the beginning of each menstruation is the early follicular stage. Each participant completed the experiments of this study during their early follicular, late follicular, and mid-luteal phases within the same menstrual cycles. In addition, in the follicular phase, participants were provided with a set of ovulation (LH) tests to be used in the middle of their cycle to determine the occurrence of ovulation. A positive result on any of the ovulation (LH) tests indicates that a woman is in the late follicular phase [29]. This was also helpful in accurately determining late follicular and mid-luteal phases. In addition, saliva samples were collected on the day of the experiment to further confirm hormone levels.

All participants had normal or corrected vision and no history of mental illness. Participants provided informed consent prior to the experiment and received a cash payment at the conclusion of the experiment. This study was approved by the Ethics Committee of Shanghai University of Sport (102772019RT004).

### 2.2. Assessment Scales

The Depression, Anxiety, and Stress Scale (DASS) [30] was completed by participants prior to the experiment and was used to assess participants’ levels of depression, anxiety, and stress on the day of their experiment. The DASS is a very reliable test for measuring depression (α = 0.95), anxiety (α = 0.90), and stress (α = 0.93). The State-Trait Anxiety Inventory (STAI-T; [31]) measured levels of state and trait anxiety in subjects. The Positive Affect and Negative Affect Scale (PANAS) [32] is a 20-item measurement method used to evaluate positive (10 items) and negative (10 items) emotions, involving the emotional level of the subject in their current state. This scale has exhibited good retest reliability and internal consistency and excellent convergence [32].

### 2.3. Experiments

#### 2.3.1. Experiment 1: Performance of the Compatible Effect of Approach–Avoidance in Women at Different Menstrual Cycle Phases during Conscious Processing

##### Materials

The experiment used 50 positive (valence: *M* = 7.27; arousal: *M* = 0.96) and 50 negative (valence: *M* = 2.73; arousal: *M* = 0.91) emotion pictures, all selected from the International Affective Picture System (IAPS) [33]. The difference in image valence between the two groups of pictures was significant (*t* (114) = 26.16, *p* < 0.001), although there was no significant difference in arousal (*t* (114) = 0.17, *p* = 0.26). An additional 6 positive and 6 negative emotional pictures were selected to serve as practice stimuli to familiarize the subjects with the experimental procedure and reaction mode. Pictures of the subjects’ faces that were collected during the experiment were discarded at the end of the experiment. All of the images were presented on a Lenovo laptop computer with a 14-inch screen (1024 × 768 resolution; 85 Hz refresh rate).

##### Procedure

The procedure was programmed using MATLAB R2016b (®MathWorks, Netdick, MA, USA)to control the manikin task. Initially, 12 emotional pictures (6 positive and 6 negative) were presented as a practice trial. Only when the correct response rate reached 95% in the practice were the participants allowed to begin the formal experiment. The specific steps of the practice trial and formal experiment are as follows. First, a cross fixation point appears in the middle of the upper or lower part of the screen (50% probability for each). After 600 ms, a picture of the subject’s face is presented at the same position as the fixation point for 750 ms. To control labeling of the reaction types, the subject’s face randomly appears in the upper or lower region of the screen, with the distribution balanced according to the experimental conditions. Then, an emotional picture is shown in the center of the screen for 2500 ms. The task directions are for the participant to press the “8” or “2” keys with the right index finger as soon as possible to accurately move the picture of their face up or down, respectively, so as to indicate a behavior of approaching or avoiding the emotional picture presented. Prior to each trial, subjects are to place their right index finger on the “5” key of the keypad to ensure that they start from the same point when pressing the “8” or “2” key. Within 500 ms after pressing the key, the self-face moves to the edge of the emotional picture or the screen edge to create a realistic approach or avoidance feeling for the participants. The self-face and picture then disappear. Pressing of the “8” or “2” key is not the action to indicate an approach or avoidance response. The participants are to simultaneously consider the position of their own face picture (whether it is up or down relative to the emotional picture) and the type of emotional picture shown (positive vs. negative), and then decide if they want to demonstrate an approach or avoidance response. The inter-trial interval was 1500 ms.

**Figure 1:**
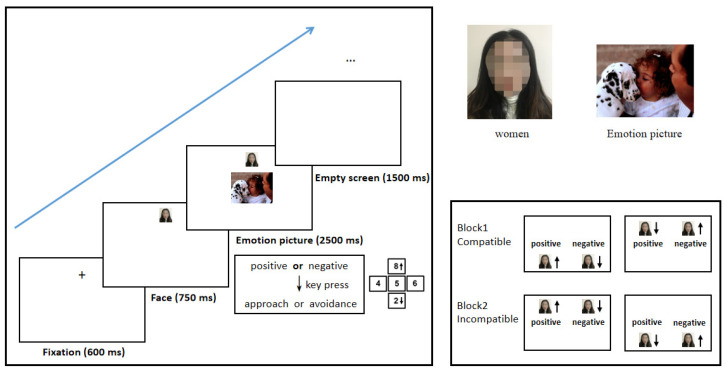
Experimental procedure in Experiment 1. A fixation point appeared either in the middle of the upper or the lower half of the screen for 600 ms. A self-face of the participant then appeared in the same position of the fixation point. At 750 ms after the appearance of the self-face, an emotional (positive or negative) picture was presented at the center of the screen (2500 ms). The self-face can be moved downward by pressing the “2” key and can be moved upward by pressing “8” key. Participants were instructed to move the self-face close to or away from the picture. All illustrations disappeared 500 ms after key press. The inter-trial interval was 1500 ms.

In the formal experiment, participants completed two blocks. Block 1 presented an evaluation-compatible block (approaching positive pictures and avoiding negative pictures) in which the subjects were asked to move their own face pictures closer to the positive emotional picture and away from the negative emotional picture, for a total of 40 trials. Meanwhile, Block 2 presented an evaluation-incompatibility block (approaching negative pictures and avoiding positive pictures), which also contained 40 trials. Each block contained all of the experimental conditions tested, and the number of trials for each experimental condition was the same. To control the order effect, half of the subjects completed Block 1 first and then Block 2, while the other half of the subjects completed Block 2 and then Block 1. In order to protect the rights and interests of the subjects, the pictures taken of the subjects’ faces for this study were destroyed immediately upon completion of the experiment. An overview of the formal experimental procedure is presented in Figure 1.

#### 2.3.2. Experiment 2: Performance of the Compatible Effect of Approach–Avoidance in Women at Different Menstrual Cycle Phases during Unconscious Processing

##### Materials

The emotional picture material used in Experiment 2 was the same as that used in Experiment 1. The material had 50 positive and 50 negative emotion pictures, and there were 25 pictures with people and 25 pictures without people for each emotion type. The same 12 emotional pictures (6 positive and 6 negative) used in Experiment 1 were also selected as the practice stimuli for Experiment 2. Each type of emotion picture was divided into half with and half without people. The pictures taken of the subjects’ faces for this experiment were destroyed immediately upon completion of the experiment.

##### Procedure

**Figure 2:**
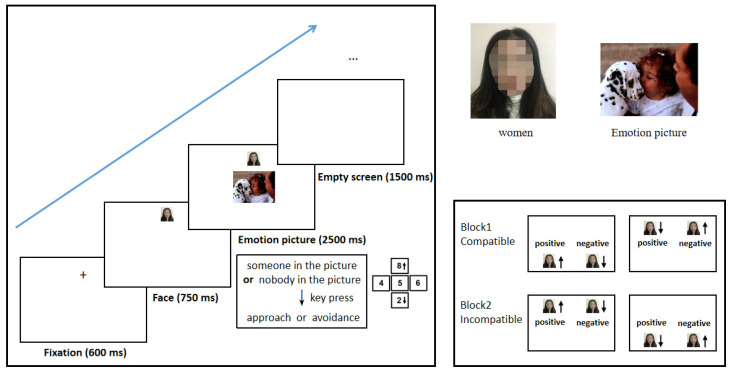
Experimental procedure in Experiment 2. The procedure used was similar to that for Experiment 1, except the subjects were required to make an approach–avoidance response based on judging whether there was a person in the picture.

The instrument used was the same as used in Experiment 1. The procedure used was similar to that for Experiment 1, except the subjects were required to make an approach–avoidance response based on cognitive classification of the picture. Thus, the participant was asked to judge whether there were people in the picture (see Figure 2). A total of 80 trials were divided into two blocks: Block 1 (approaching pictures that contain people and avoiding pictures that do not), including 10 positive emotion without person pictures, 10 positive emotion with person pictures, 10 negative emotion without person pictures, and 10 negative emotion with person pictures, a total of 40 trials. Block 2 (approaching pictures that do not contain people and avoiding pictures that do) used the remaining 40 images for a total of 40 trials.

### 2.4. Study Process

Participants were first asked to rinse their mouth with warm water and then wait 10 min. Disposable pipettes and Corning cryo-storage tubes (3 mL) were used to collect saliva. Each subject collected two tubes of saliva each time, and the concave level of saliva volume in each tube had to be above the 2 mL mark [34]. In order to ensure the continuous activity of saliva, the freshly collected saliva samples were frozen at −20 °C immediately after collection and stored until the assay.

Participants sat comfortably 60 cm from the screen in a room electrically shielded, air-conditioned, and dimly lit. In order to assess emotional state, participants completed the Positive Affect and Negative Affect Scale (PANAS) before the experiment. They also completed the Depression, Anxiety and Stress Scale (DASS) [30] to assess the level of depression, anxiety and stress on the test day. The State-Trait Anxiety Scale (STAI-T; [31]) measured the level of state and trait anxiety of the subjects. After that, the subjects began to conduct a formal improved “Manikin Task”, during which the subjects were asked to minimize head movement, blinking, mind wandering and other behaviors. MATLAB software was used to automatically record the response time of the subjects.

### 2.5. Hormonal Analyses of Saliva Samples

Estradiol and progesterone levels were measured in three saliva samples from each participant collected. These samples were also used to confirm the participants’ menstrual cycle status. Salivary Estradiol (SLV-4188) and Salivary Progesterone (SLV-2931) enzyme-linked immunosorbent assay (ELISA) kits were used (DRG^®^, Marburg, Hessen, Germany). According to the instructions for the kits, saliva samples can be stored at temperatures ranging from 2 to 8 °C for up to 7 days without affecting the validity of the test. If saliva samples are stored at −20 °C, they can be stored for longer. It is also indicated that repeated freezing and thawing will not affect the validity of the tests. Sensitivity of the DRG kit was 0.4 pg/mL for estradiol and 3.9 pg/mL for progesterone at 95% confidence intervals. For detection of estradiol, the in-box confidence level of the kit was 2.1–3.8%, and the inter-box confidence level was 2.6–6.9%. For progesterone, the in-box and inter-box confidence levels were 5.3–7.7% and 4.7–7.6%, respectively. The kits also reached the standard of accuracy within 15%. Hormone testing for this study was performed at the Beijing Protein Innovation Co., Ltd., in China.

### 2.6. Data Analysis

Data were analyzed with SPSS 16.0 software (Chicago, IL, USA). One-way analysis of variance (ANOVA) was used to analyze the scale scores and estradiol and progesterone levels in saliva across the cycle phases. We conducted 2 (emotion: positive, negative) × 2 (motivational behavior: approach, avoidance) × 3 (cycle: early follicular, late follicular, mid-luteal phases) repeated-measures ANOVA for the reaction time data. The degrees of freedom of the repetition factor were corrected according to the Huynh–Feldt method. Significant main effects and interactions were subjected to post hoc tests using the Bonferroni correction and simple effects analyses. As a measure of effect size, partial eta square ($\eta_{p}^{2}$) was reported when effects were compared. Greenhouse–Geisser corrected *p* values were used for the ANOVA calculations. *p* values less than 0.05 were considered significant. Relationships between sex hormone levels and the reaction time of the compatibility effect of approach–avoidance were examined with the Pearson Correlation.

## 3. Results

### 3.1. Subjective Measures

The PANAS scale showed that menstrual cycle status did not affect positive emotion (*F*(2, 78) = 0.332, *p* = 0.718) or negative emotion (*F*(2, 78) = 0.627, *p* = 0.537) responses. DASS scale results also showed no significant differences in stress (*F*(2, 78) = 1.733, *p* = 0.183), anxiety (*F*(2, 78) = 0.453, *p* = 0.638), or depression (*F*(2, 78) = 1.076, *p* = 0.346) among the three phases of the menstrual cycle examined. STAI scores further showed no significant cycle effect (state anxiety (*F*(2, 78) = 0.128, *p* = 0.88), trait anxiety (*F*(2, 78) = 0.982, *p* = 0.379)).

### 3.2. Hormone Levels

**Figure 3:**
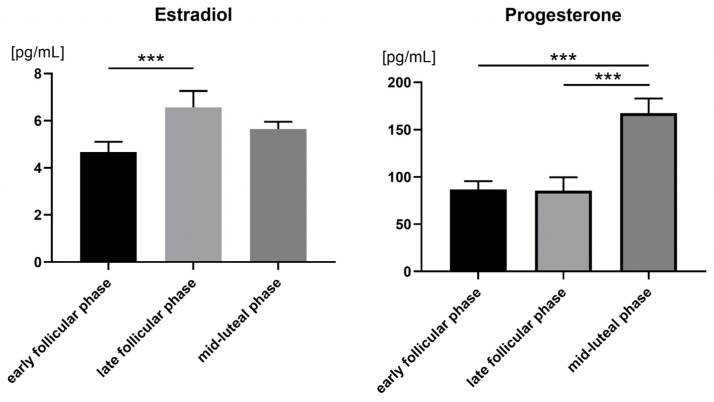
Levels of estradiol and progesterone in saliva, across the menstrual cycle. Error bars represent the standard error of the mean. *** *p* < 0.001.

Figure 3 presents the effects of the menstrual cycle phase on hormones. The estrogen levels detected during early follicular, late follicular, and mid-luteal phases of the menstrual cycle showed significant differences (estrogen: *F*(2, 78) = 4.629, *p* = 0.013; progesterone: *F*(2, 78) = 12.850, *p* = 0.000). The level of estradiol in the late follicular phase was significantly higher than in the early follicular (*p* = 0.018). Similarly, the level of progesterone at the mid-luteal phase was significantly higher than in the early follicular (*p* = 0.000) and late follicular (*p* = 0.000) phases.

### 3.3. Performance

#### 3.3.1. Reaction Times in Experiment 1

**Figure 4:**
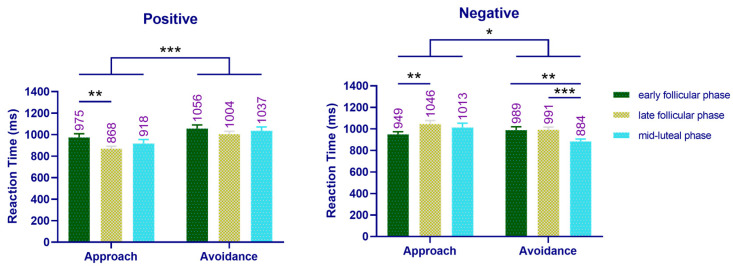
Reaction time of the improved “manikin task” across the cycle phases in Experiment 1. Left: the reaction time of women performing approach–avoidance behavior to a positive emotional picture across the cycle phases. Right: the reaction time of women performing approach–avoidance behavior to a negative emotional picture across the cycle phases. Mean RT above bar. Error bars represent the standard error of the mean. *** *p* < 0.001; ** *p* < 0.01; * *p* < 0.05.

The main effect of motivation type was significant (*F*(1, 26) = 12.414, *p* = 0.002, $\eta_{p}^{2}$ = 0.323), and the response time of approach (962 ms) was significantly shorter than that for avoidance (994 ms) (*p* = 0.002). The interaction between menstrual cycle and emotion type was significant (*F*(2, 52) = 26.166, *p* = 0.000, $\eta_{p}^{2}$ = 0.502). A simple effect analysis showed that women in late follicular phase (936 ms) exhibited shorter response times to positive images than women in early follicular phase (1016 ms) (*p* = 0.008). Similarly, women in mid-luteal (949 ms) and early follicular (969 ms) phases had shorter responses time to negative images than women in late follicular phase (1019 ms) (*p* = 0.005). The interaction between emotion type and motivation type was significant (*F*(1, 26) = 25.806, *p* = 0.000, $\eta_{p}^{2}$ = 0.498). A simple effect analysis showed that the approach response (920 ms) was significantly faster than the avoidance response (1033 ms) to positive stimuli (*p* = 0.000), while the avoidance response to negative stimuli (955 ms) was significantly faster than the approach response (1003 ms, *p* = 0.022), as shown in Figure 4. The interaction of menstrual cycle, mood type, and motivation type was also significant (*F*(2, 52) = 7.994, *p* = 0.001, $\eta_{p}^{2}$ = 0.235). A simple effect analysis showed that women in late follicular phase (868 ms) approached positive stimuli significantly faster than women in early follicular phase (975 ms) (*p* = 0.004). When asked to approach negative stimuli, women in late follicular phase (1046 ms) exhibited a significantly longer conflict time than women in early follicular phase (949 ms) (*p* = 0.001). When the task was to avoid negative stimuli, the response time of women in mid-luteal phase (884 ms) was significantly shorter than women in early follicular phase (989 ms) (*p* = 0.003) or late follicular phase (991 ms) (*p* = 0.000).

#### 3.3.2. Correlation between Reaction Time and Sex Hormone Levels in Experiment 1

**Figure 5:**
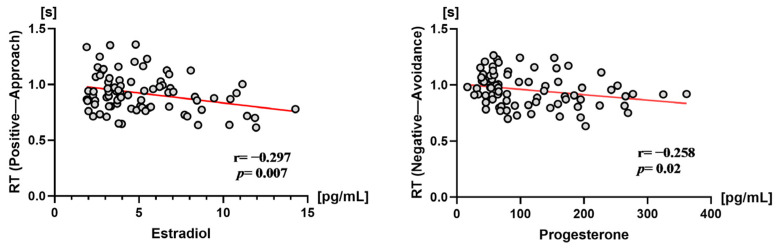
Correlation scatter plots between sex hormone levels (**left**: estradiol; **right**: progesterone) and reaction time in Experiment 1.

Estradiol level negatively correlated with response time to approaches of positive stimuli (*r* = −0.297, *p* = 0.007). Similarly, progesterone level negatively correlated with response time to avoidance of negative stimuli (*r* = −0.258, *p* = 0.020). As shown in Figure 5.

#### 3.3.3. Response Times in Experiment 2

**Figure 6:**
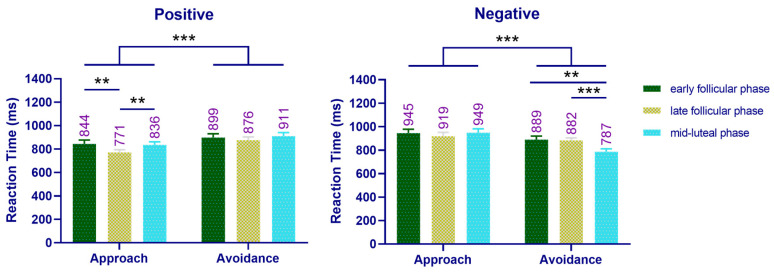
Reaction time of the improved “manikin task” across the cycle phases in Experiment 2. Left: the reaction time of women performing approach–avoidance behavior to a positive emotional picture across the cycle phases. Right: the reaction time of women performing an approach–avoidance behavior to a negative emotional picture across the cycle phases. Mean RT above bar. Error bars represent the standard error of the mean. *** *p* < 0.001; ** *p* < 0.01.

The main effect of emotion type was significant (*F*(1, 26) = 35.087, *p* = 0.000, $\eta_{p}^{2}$ = 0.574), and the response time to a positive stimulus (856 ms) was significantly shorter than that to a negative stimulus (895 ms) (*p* = 0.000). The interaction between menstrual cycle and emotion type was also significant (*F*(2, 52) = 15.468, *p* = 0.000, $\eta_{p}^{2}$ = 0.373). A simple effect analysis showed that presentation of positive images resulted in a shorter response time for women in late follicular phase (824 ms) compared with women in early follicular (871 ms) (*p* = 0.042) or mid-luteal (874 ms) (*p* = 0.015) phases. The interaction between emotion type and motivation type was significant (*F*(1, 26) = 53.952, *p* = 0.000, $\eta_{p}^{2}$ = 0.675). A simple effect analysis showed that the approach response (817 ms) was significantly faster than the avoidance response (895 ms) to positive pictures (*p* = 0.000), while the avoidance response (853 ms) was significantly faster than the approach response (937 ms) to negative pictures (*p* = 0.000) (Figure 6).

Regarding menstrual cycle and motivation type, a significant interaction was observed (*F*(2, 52) = 15.552, *p* = 0.000, $\eta_{p}^{2}$ = 0.374). In terms of avoidance response, women in mid-luteal phase (849 ms) exhibited a shorter response time than women in late follicular phase (879 ms) (*p* = 0.038). In addition, the triad interaction of menstrual cycle, emotion type, and motivation type was found to be significant (*F*(2, 52) = 3.407, *p* = 0.041, $\eta_{p}^{2}$ = 0.116). A simple effects analysis showed that women in late follicular phase (771 ms) exhibited a significantly shorter time to approach positive stimuli than women in early follicular phase (844 ms) (*p* = 0.005) or mid-luteal phase (836 ms) (*p* = 0.001). When negative stimulus avoidance was required, the response time of women in mid-luteal phase (787 ms) was significantly shorter than that of women in early follicular phase (889 ms) (*p* = 0.002) or late follicular phase (882 ms) (*p* = 0.000) (Figure 6).

#### 3.3.4. Correlation between Response Times and Sex Hormone Levels in Experiment 2

Detected levels of estradiol and progesterone did not significantly correlate with time of approach–avoidance (*p* < 0.05).

## 4. Discussion

In this study, we investigated the influence of women’s menstrual cycle on approach–avoidance behaviors during conscious (Experiment 1) and unconscious (Experiment 2) processing of emotional stimuli. We also examined a potential role for ovarian hormones in approach–avoidance-motivated behaviors. With the use of an improved “manikin task” paradigm, the approach response to positive stimuli was found to be significantly faster than the avoidance response. Conversely, the avoidance response to negative stimuli was significantly faster than the approach response. These results support the hypothesis of the Motivational Orientation Theory that emotional stimuli can activate approach–avoidance motivations consistent with stimulus valence, without the stimulus valence being explicitly processed. In addition, the menstrual cycle was found to affect the motivational behaviors of women in response to emotional stimuli. For example, women in late follicular phase were found to be significantly more inclined to approach positive stimuli than women in early follicular phase. Meanwhile, women in mid-luteal stage performed motivational responses to avoid negative stimuli faster than women in late follicular phase. These results are consistent with the hypothesis of the Motivational Orientation Theory, and they indicate that women’s motivational processing of emotional information is affected by menstrual cycle. Thus, ovarian hormones are not only related to women’s emotional sensitivity but can also potentially affect the ability of women to seek advantages and avoid harms in response to information from their environment. The current results are discussed below in the context of previous literature.

### 4.1. The Approach–Avoidance Performance of Females in a Conscious/Unconscious State

When female subjects consciously processed the valence of emotional pictures, they exhibited a faster approach response to positive stimuli and a faster avoidance response to negative stimuli. These observations are consistent with the “compatible effect of approach–avoidance”. Moreover, this conclusion is consistent with previous research [2,3,4,5]. As mentioned above, the main difference between the Motivational Orientation Theory and the TEC lies in whether emotional valence is processing specific, and whether it can automatically activate compatible approach–avoidance behavior without relying on explicit emotional evaluations [8]. The results of Experiment 2 show that when an approach–avoidance response was elicited from participants based on their cognitive classification of an emotional stimulus (e.g., judging whether there was a person in the picture), the subjects processed the valence of the emotional picture unconsciously. Thus, even when cognitive classification was used as a masking stimulus for the valence judgment of an emotional picture, the “compatible effect of approach–avoidance” was maintained. Therefore, the two experimental designs employed in the present study help resolve the dispute between the Motivational Orientation Theory and the TEC, and our results support the Motivational Orientation Theory.

### 4.2. Influence of the Menstrual Cycle on Approach–Avoidance Behavior of Women

#### 4.2.1. Analysis of the Characteristics of Approach–Avoidance Behavior in Women in Late Follicular Phase

In Experiments 1 and 2, women in late follicular phase were significantly more likely to approach positive stimuli than women in early follicular phase during both conscious and unconscious processing of emotional information. Moreover, during unconscious processing, women in late follicular phase were also significantly more likely to approach positive stimuli than those in mid-luteal phase.

Dreher (2007) et al. found that both expected and actual monetary rewards caused higher activation of the reward system in mid-follicular, and this response was related to estrogen levels [17]. This result helps explain the observation in the present study that women in late follicular phase approached positive stimulation more quickly than they did during other phases of the menstrual cycle. Thus, in the late follicular phase, women experience stronger approach motivation, they activate the behavioral approach system more effectively, and they implement approach behavior. In terms of hormone secretion, estrogen secretion peaks during the late follicular phase. Accumulating evidence indicates that estrogen may temporarily enhance stimulated dopamine release [35,36] and synthesis [37]. Given that the behavioral approach system is closely related to the dopaminergic pathway [38], it is possible that women are more likely to experience pleasurable feelings in the late follicular phase due to increased secretion of dopamine, and this mobilizes the behavioral approach system to implement approach behavior. This is consistent with estrogen and progesterone secretion levels acting in combination with other complex physiological and psychological factors to contribute to cycle specificity of female approach–avoidance behavior.

In Experiment 1, it was observed that women in the late follicular phase experienced significantly longer conflict when considering approaching negative stimuli than women in the early follicular phase. It has been reported that women’s ability to recognize angry expressions declines during the late follicular phase when the level of estrogen is higher [39]. This may indicate that women in the late follicular phase are more likely to feel and approach positive emotions related to reward. Accordingly, women may have greater emotional conflict when asked to approach negative stimuli in the late follicular phase, and this may manifest as a significantly slower approach speed. Thus, ovulation is more likely to be a period during which women experience feelings of happiness and attractiveness, and it is supported by many studies [40,41].

#### 4.2.2. Relationship between Estradiol Levels and Benefit–Avoidance Behavior in Women in Late Follicular Phase

The role of estradiol in mediating the psychological behavior of women has been examined by comparing women in early (low estradiol) versus late (high estradiol) follicular phases. In Experiment 1 of the present study, the women in late follicular phase were significantly more inclined to approach positive stimuli than women in the early follicular phase under conscious processing conditions. A Pearson correlation analysis further showed that levels of estradiol significantly and negatively correlated with the response time for approaching positive stimuli (*r* = −0.297, *p* = 0.007). It showed that women in the late follicular phase take less time to approach positive stimulation than women in the early follicular phase due to their higher levels of estrogen. It was previously demonstrated that injections of estrogen can reduce depressive symptoms in postmenopausal women [14]. Thus, it is possible that estrogen may play an important role in how women experience positive emotions and engage in motivational behavior toward favorable things. This may represent the “source of happiness” and “action power” of women that is observed.

We observed that the longer response times in approaching negative stimuli by women in late follicular phase did not correlate with estrogen levels (*r* = −0.096, *p* = 0.393). Estrogen level only correlated with behavioral response under conditions where the participants were close to positive stimuli. In contrast, estrogen level was not related to the conflict behavioral responses experienced when the participants were close to a negative stimulus. In addition, the results of Experiment 2 demonstrate that there is no significant correlation between estradiol level and the approach–avoidance response when unconscious processing conditions were investigated. These results indicate that an association between estradiol level and positive approach response is affected by conscious processing of emotional stimuli. This close relationship occurs when people process explicit emotions, yet it does not exist during unconscious processing.

#### 4.2.3. Analysis of Approach–Avoidance Behavior Characteristics in Women in Mid-Luteal Phase

Women in mid-luteal phase exhibited significantly shorter avoidance response times to negative stimuli under both conscious and unconscious processing states compared with women in early or late follicular phases. These results are consistent with those of previous studies that suggest that the luteal phase of the menstrual cycle enhances the sensitivity of women to threatening stimuli in the environment [21,42]. For example, Masataka and Shibasaki (2012) observed that women in the luteal phase had shorter responses to threatening stimuli (e.g., snakes, tigers, and other predators) than during the early and late follicular phases. However, when faced with neutral stimuli (such as plants), no difference in response time between the different menstrual cycles was observed [21]. Furthermore, researchers have found that during the luteal phase, the physical changes that take place in a woman’s body in preparation for a possible pregnancy are accompanied by psychological changes caused by the same hormonal fluctuations. Observed psychological changes include increased social surveillance [43], increased vigilance against physical threats [21], sensitivity to infectious agents [27,44], etc. These psychological changes can subsequently lead to approach–avoidance behavior. This strong motivation to avoid harm during the luteal phase is also related to protecting one’s own health. During the luteal phase, the immune system is suppressed to prevent the body from rejecting the developing fetus, which in turn leads to increased susceptibility to infection [45]. Thus, people tend to take precautions against any possible source of infection, while exhibiting increased sensitivity to nausea stimuli [44,46]. A similar mechanism may account for the prevalence of food aversion during pregnancy [47].

#### 4.2.4. Relationship between Progesterone Level and Approach–Avoidance Behavior in Women in Mid-Luteal Phase

A correlation analysis between progesterone level and approach–avoidance behavior showed that levels of progesterone negatively correlated with reaction times for avoiding negative stimuli. This observation suggests that greater secretion of progesterone leads to a shorter response time in avoiding negative stimuli. Correspondingly, women in mid-luteal phase avoid negative stimuli more quickly than women in other phases of the menstrual cycle. Klatzkin et al. (2006) previously demonstrated that exogenous injections of progesterone can increase levels of negative emotions in women in the early follicular phase and in women experiencing menopause [15]. Therefore, the results of the present study further confirm that high levels of progesterone may not only enhance women’s negative emotional experiences but may also be related to women’s further enhancement of avoidance-motivated behavior in response to negative stimuli. Thus, a strong relationship between progesterone and the acquisition and response of negative emotions appears to exist.

However, while Experiment 2 did not show a significant correlation between levels of progesterone and an avoidance of negative stimulus response, an association between progesterone level and negative avoidance response was found to be affected by conscious processing of emotional stimuli. For example, a close association was observed under the condition of explicit emotion processing, yet one was not observed under the condition of unconscious processing.

### 4.3. Limitations and Strengths

This study has filled some gaps in the research about the relationship between female emotions and sex hormones, but there are still some shortcomings that deserve to be improved in further research. First, the secretion of sex hormones is affected by many aspects such as physiology and psychology, and some subjects were excluded because their menstrual cycle was delayed or advanced for various reasons. We finally selected a relatively moderate sample size, which has enough power, but the sample size still needs to be increased as much as possible to verify the universality and reliability of the results. In addition, not only estradiol and progesterone, but also prolactin and oxytocin, etc., can vary with the menstrual cycle, and both are also increased in the late follicular phase [48]. Whether these two hormones affect the participants’ performance of approach–avoidance needs to be further investigated in future studies. Finally, since the current study is still limited to the behavioral domain, future studies could explore the underlying neural mechanisms using event-related potentials or fMRI.

## 5. Conclusions

The behavioral evidence presented in this study demonstrates that the menstrual cycle of women affects their psychological and motivational behaviors in seeking advantages and avoiding disadvantages. Specifically, women in late follicular phase approach positive stimuli more quickly, while women in mid-luteal phase avoid negative stimuli more acutely, which exist in both conscious and unconscious processes. It is recognized that differences in female approach–avoidance behaviors are related to levels of estrogen and progesterone that are secreted at various stages of the menstrual cycle. Consequently, sex hormones have important roles in mediating emotionally motivated behaviors, including female approach–avoidance behavior. During different menstrual cycle phases, emotional state, other hormone secretion changes, and immune system mechanisms lead to changes in the body’s self-protection motivation. These may also contribute to the differences in approach–avoidance behaviors observed in women. In the present study, the “compatible effect of approach–avoidance” was observed in both conscious and unconscious processing of emotional pictures, thereby supporting the Motivational Orientation Theory. Therefore, this study provides valuable insight into an active area of research regarding the relationship between sex hormones and motivational behavior. By characterizing the contributions of the menstrual cycle to women’s ability to maximize self-interest and avoid risk, women are better able to understand and improve themselves.

## Data Availability

The datasets generated for this study are available on request to the corresponding author.
